# Shaping visual space perception through bodily sensations: Testing the impact of nociceptive stimuli on visual perception in peripersonal space with temporal order judgments

**DOI:** 10.1371/journal.pone.0182634

**Published:** 2017-08-04

**Authors:** Lieve Filbrich, Andrea Alamia, Séverine Blandiaux, Soline Burns, Valéry Legrain

**Affiliations:** 1 Institute of Neuroscience, Université catholique de Louvain, Brussels, Belgium; 2 Faculty of Psychology and Educational Sciences, Université catholique de Louvain, Louvain-la-Neuve, Belgium; Centre de neuroscience cognitive, FRANCE

## Abstract

Coordinating spatial perception between body space and its external surrounding space is essential to adapt behaviors to objects, especially when they are noxious. Such coherent multisensory representation of the body extended into external space is conceptualized by the notion of peripersonal reference frame, mapping the portion of space in which somatic and extra-somatic inputs interact closely. Studies on crossmodal interactions between nociception and vision have been scarce. Here we investigated how the perception of visual stimuli, especially those surrounding the body, can be impacted by a nociceptive and potentially harmful stimulus inflicted on a particular body part. In two temporal order judgment tasks, participants judged which of two lateralized visual stimuli, presented either near or far from the body, had been presented first. Visual stimuli were preceded by nociceptive stimuli, either applied unilaterally (on one single hand) or bilaterally (on both hands simultaneously). In Experiment 1 participants’ hands were always placed next to the visual stimuli presented near the trunk, while in Experiment 2 they could also be placed next to the visual stimuli presented far from the trunk. In Experiment 1, the presence of unilateral nociceptive stimuli prioritized the perception of visual stimuli presented in the same side of space as the stimulated hand, with a significantly larger effect when visual stimuli were presented near the body than when presented farther away. Experiment 2 showed that these visuospatial biases were related to the spatial congruency between the hand on which nociceptive stimuli were applied and the visual stimuli, independently of the relative distance of both the stimulated hand and the visual stimuli from the trunk. Indeed, nociceptive stimuli mostly impacted the perception of the closest visual stimuli. It is hypothesized that these crossmodal interactions may rely on representations of the space directly surrounding specific body parts.

## 1. Introduction

For any living organism, it is important to monitor the space surrounding the body in order to avoid stimuli that have the potential to inflict damage on the body. Pain represents the archetype of physical threat. It is an unpleasant sensory and emotional experience that acts as a warning signal about potential body damage, with the aim of triggering behaviors purposely oriented to defend or restore the physical integrity of the body. Pain is initiated, in normal conditions, by the activation of specific sensory receptors, the nociceptors, characterized by the ability to code high intensity, and, as a consequence, potentially harmful stimuli. In order to adapt the behavior to possibly harmful stimuli, we need to detect and localize the part of the body that is potentially being harmed, i.e. the body part on which the nociceptive stimulus is applied. The spatial position of sensory information can be coded according to different frames of reference, i.e. coordinate systems. Localizing the position of a nociceptive stimulus depends partly on projections of spatially organized sensory receptor fields on the body surface to specific spatially segregated groups of neurons in the cortex [1–4]. This somatotopic frame of reference, representing the skin surface anatomically, is however not sufficient to respond adequately to the potential threat. Localizing the position of the possibly harming object in external space is also of primary importance, in order to spatially guide defensive motor responses. It is therefore necessary to coordinate the representation and perception of the space of the body and its surrounding external space. Such a coherent multisensory representation of the body space and its surrounding space is conceptualized by the notion of peripersonal reference frames. The peripersonal reference frames are mapping systems coding the portion of space in which somatic and extra-somatic (e.g. visual) stimuli can interact closely [5–9]. The existence of peripersonal frames of reference has largely been documented for touch [10–13], but very poorly investigated for nociception and pain [14]. However, studying the involvement of a peripersonal frame of reference in localizing nociceptive stimuli is not only important to apprehend how nociception is integrated with other sensory information to build a full representation of physical threats, but also to broaden the understanding of chronic pain pathophysiology. Indeed, it has recently been suggested that chronic pain can impair the representation and the perception of external space, although the exact nature of these impairments is still unknown [15]. Interactions between nociception and other sensory inputs have been demonstrated in studies showing that the level of pain evoked by a nociceptive stimulus can depend on the posture and the vision of the body part on which the nociceptive stimulus is applied [16–20] (however, see [21, 22]). Similarly, Sambo et al. [23] and De Paepe et al. [24] showed that judgments about the occurrence of nociceptive stimuli depend on the relative position of the limbs. In both studies a temporal order judgement (TOJ) task was used (see [25]), and participants had to judge which of two nociceptive stimuli, one applied on either hand, was perceived as being presented first. The task was performed with the hands either in a normal, uncrossed posture or with the hands crossed over the body midline. In studies on the spatial perception of touch, the crossed posture is known to reveal a competition between somatotopic and spatiotopic reference frames, indexed by a deterioration of performance when the hands are placed in a crossed posture (see [26]). Similarly, TOJs on nociceptive stimuli were also affected by the crossed posture, suggesting that space-based reference frames are also used to code nociceptive stimuli [23, 24]. Even more compelling evidence was provided by De Paepe et al. [27] who showed, also using TOJ tasks, that judgments about nociceptive perception were systematically biased by the presence of a visual stimulus shortly preceding the nociceptive stimuli in the same side of space. This visual cue facilitated indeed the perception of the nociceptive stimulus applied on the ipsilateral hand, to the detriment of the nociceptive stimulus applied on the opposite hand. Importantly, this effect of the visual cue on the perception of the nociceptive stimuli was most pronounced when the visual cue was presented close to the participant’s hand, as compared to conditions in which the cue was presented farther away. They demonstrated furthermore that the effect of this close proximity between visual and nociceptive stimuli was independent of the relative posture of the body limb [24]. Finally, De Paepe et al. [28] showed that reaction times to nociceptive stimuli were differently influenced by the vision of a visual stimulus approaching the to-be-stimulated hand (that is, a stimulus that could possibly contact the body) vs. a receding visual stimulus (that is, a stimulus that will not contact the body).

If these studies demonstrated that the detection, the localization and the perception of a nociceptive stimulus depends on the perception of a visual stimulus that may have an impact on the body, it is also highly relevant to investigate the reverse link, that is, how the perception of visual stimuli around the body can be affected by a nociceptive and potentially harmful stimulus inflicted on a particular body part. In non-human primates, such links are thought to be based on, at least for innocuous tactile stimuli, the existence of multimodal neurons, that is, neurons able to respond to sensory inputs from different sensory modalities (see [29]). For instance, Rizzolatti and colleagues [7, 8] discovered neurons in the ventral premotor cortex (PMv) responding to tactile stimuli applied on a given body part, but also to visual stimuli. Importantly, for some of these PMv neurons, the visual receptive fields are limited to the portion of external space immediately adjacent to the associated tactile receptive fields [7]. Moreover, the receptive fields of these neurons do not move with eye movement, but with movements of the limb to which they are anchored [30–33]. Similar neurons have been found in the ventral intraparietal sulcus (VIP), with the difference that their visual receptive fields are mostly head-centered [34, 35]. In other words, as far as visuospatial perception is concerned, the function of such bimodal neurons would be to remap the location of visual inputs from a retinotopic to a spatiotopic frame of reference, but, crucially, by using body parts as coordinate references. Finally, direct electrical stimulation of neurons in the PMv and VIP elicit defensive motor reactions in the monkeys [36, 37], similar to those evoked by direct application of unpleasant somatosensory stimuli on the monkeys’ skin [37], suggesting that such neurons could be involved in coding threat information [29]. However, up to now, there is only one study that demonstrated the existence of neurons in the inferior parietal lobe, close to the secondary somatosensory area, coding both thermo-nociceptive inputs as well as visual inputs that approach the body part on which the nociceptive stimulus is applied [38].

In the present studies we investigated, in humans, how the perception of the visuospatial environment can be impacted by bodily sensations, especially when these sensations are conveyed through the nociceptive system, that is, the neural system coding and transmitting sensory information about potentially harmful somatosensory events. We hypothesized that applying a nociceptive stimulus would particularly influence the perception of visual stimuli surrounding the body, that is, stimuli that might have an immediate impact on the body, in order to prioritize their processing over stimuli located at a farther distance. To this aim, we used two TOJ tasks, similar to those used by De Paepe and colleagues [24, 27] but with pairs of visual stimuli, one stimulus presented in each side of space. In a first experiment, visual stimuli were presented either near or far from the body, using its main axes, such as the trunk, as a reference, and the participants’ hands were placed next to the near visual stimuli. Visual stimuli were shortly preceded by nociceptive stimuli, which were either applied unilaterally on one hand or bilaterally on both hands simultaneously. We hypothesized that the unilateral nociceptive stimuli would positively bias the perception of the visual stimuli presented in the same side of space as the stimulated hand, especially when these visual stimuli were presented near the body. In the second experiment, we added a condition during which participants were asked to place their hands next to the visual stimuli that were presented farther away from the body. This condition was aimed to disentangle whether nociceptive-visual interactions mainly rely on a spatial representation of the body as a whole or on a spatial representation restricted to the particular limb on which the nociceptive stimulus is applied. Indeed, as animal studies revealed that the visual receptive fields of multimodal neurons can be anchored to particular body parts, we hypothesized that nociceptive stimuli would mostly impact the perception of visual stimuli in the immediate proximity of the hand on which the nociceptive stimuli were applied, independently of the distance of the visual stimuli from the trunk and the relative position of the limbs. The results confirmed our hypotheses and strongly support the hypothesis that the perception of visual space, especially that of peripersonal space, can be shaped by nociceptive information.

## 2. Materials and methods

### 2.1 Experiment 1

#### 2.1.1 Participants

Twenty participants in total were recruited between February and March 2015 through advertisements on the internet. Two participants dropped out before the testing session and 18 participants finally participated in the experiment. Testing took place between March and April 2015 at the Institute of Neuroscience of the Université catholique de Louvain, Brussels. One participant was excluded before data analysis because of difficulties in perceiving the nociceptive stimuli. The mean age of the remaining 17 participants (9 women) was 23.29 ± 3.1 years (range: 19–32 years). Exclusion criteria were non-corrected vision deficits, neurological, psychiatric, cardiac or chronic pain problems, regular use of psychotropic drugs, as well as a traumatic injury of the upper limbs within the six months preceding the experiment. Participants were asked to not use any analgesic substances (e.g. NSAIDs or paracetamol) within the 12 hours preceding the experiment and to sleep at least 6 hours the night before the experiment. According to the Flinders Handedness Survey (Flanders) [39], all of the participants were right-handed. The experimental procedure was approved by the local ethic committee (Commission d’Ethique Biomédicale Hospitalo-Facultaire de l’Université catholique de Louvain) in agreement with the latest version of the Declaration of Helsinki and was carried out in accordance with the corresponding guidelines and regulations. All participants signed a consent form prior to the experimental session. Participants received financial compensation for their participation.

#### 2.1.2 Stimuli and apparatus

Nociceptive stimuli were applied at the hand dorsum using intraepidermal electrical stimulation (IES) (DS7 Stimulator, Digitimer Ltd, UK) with stainless steel concentric bipolar electrodes (Nihon Kohden, Japan; [40]). These electrodes consist of a needle cathode (length: 0.1 mm, Ø: 0.2 mm) surrounded by a cylindrical anode (Ø: 1.4 mm). They were gently pressed against the skin to insert the needle in the epidermis of the sensory territory of the superficial branch of the radial nerve. For each of the participants’ hands, absolute detection thresholds to a single 0.5 ms square-wave pulse were determined using a staircase procedure [41]. The intensity of the electrical stimulation was then individually set to twice the absolute detection threshold to selectively activate nociceptors, with a limit of 0.5 mA [42]. If necessary, these intensities were individually adapted to guarantee that stimulus intensities were perceived as equivalent for both hands (see [43] for details). Using this specific procedure, IES has been shown to selectively activate Aδ nociceptors without co-activation of Aβ mechanoreceptors [42, 44, 45]. During the experiment, stimuli consisted of trains of three consecutive 0.5 ms square-wave pulses separated by a 5 ms interpulse interval, to increase the intensity of perception while preserving the selectivity for nociceptors [44, 45]. Participants described the sensation as pricking but not necessarily as painful.

Visual stimuli were presented by means of four white light emitting diodes (LEDs) with a 17 lm luminous flux, a 6.40 cd luminous intensity, and a 120° visual angle (GM5BW97330A, Sharp Corporation, Japan). They were perceived as brief flashes. Before the experimental session participants were asked to report the position of the flashing LED (e.g. the left one in front, the right one in the back, etc.) to ensure the visibility of the LEDs. A yellow LED (min. 0.7 cd luminous intensity at 20 mA, 120° viewing angle) served as fixation point.

#### 2.1.3 Procedure

**Fig 1:**
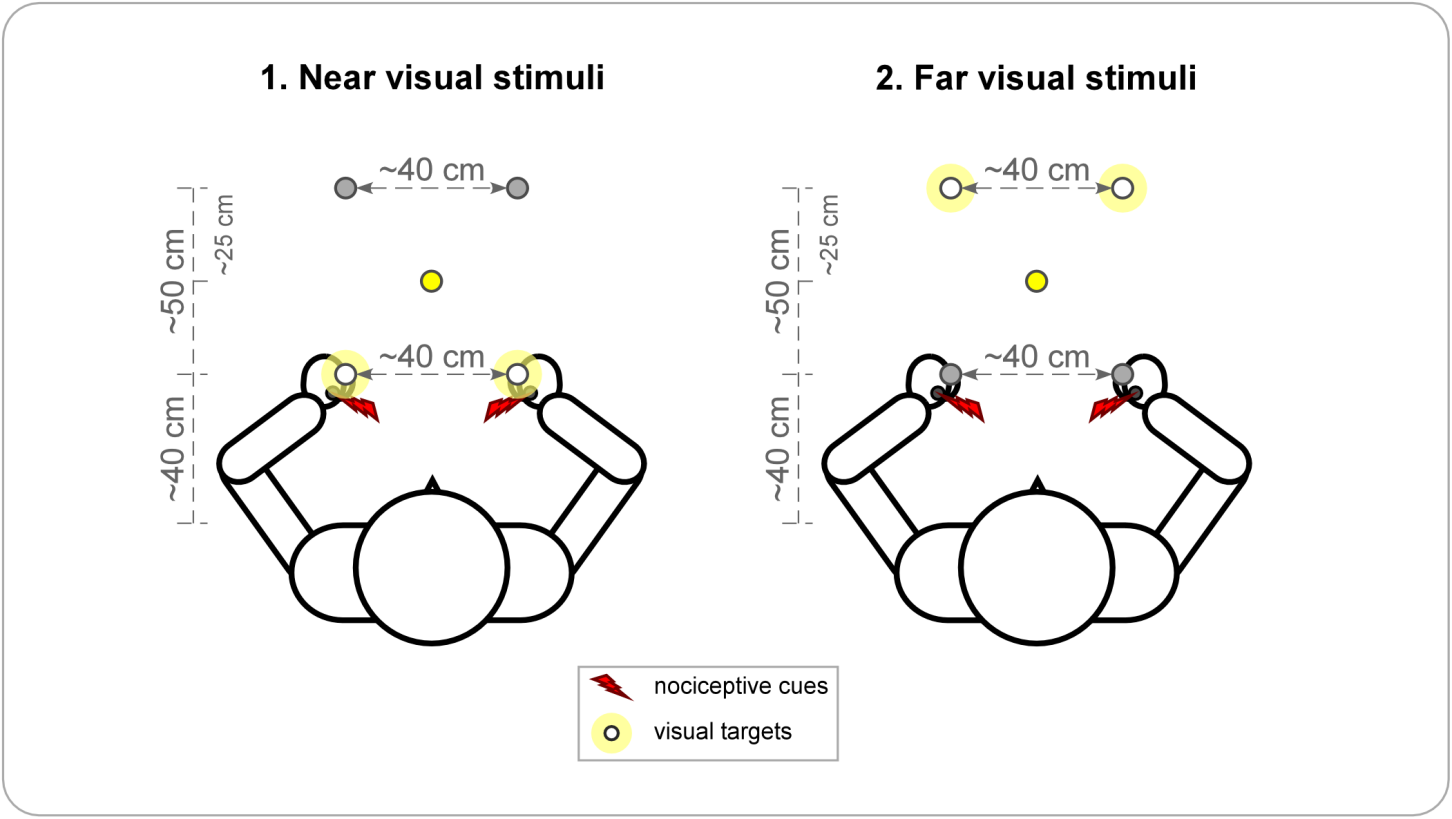
Design of Experiment 1. Visual stimuli are presented by means of two pairs of LEDs, one placed near and the other placed far from the trunk. The participants’ hands are positioned close to the near LEDs. In each block, participants perform the task only with one pair of LEDs, either *near* (1) or *far* (2) from the trunk. The task-relevant pair is considered as the visual targets and illustrated by the white circles with a slight yellow halo. Nociceptive stimuli are illustrated by the red flashes and, depending on the trial, they are either applied unilaterally on the left or the right hand or bilaterally, on both hands simultaneously. A centrally-placed yellow LED, represented by the yellow circles, serves as fixation point.

Participants were sitting in front of a table in a dimly-illuminated testing room, with their arms placed on the table and their palms down. Their heads were stabilized with a chin-rest placed ~10 cm from the trunk, in order to minimize head movements. The four white LEDs were fixed on the table. A first pair of LEDs was placed at a distance of ~40 cm from the participants’ trunk (near visual stimuli), the other pair at a distance of 50 cm from the near LEDs, and therefore at a distance of ~90 cm from the participants’ trunk (far visual stimuli) (Fig 1). For each pair, one LED was placed to the participant’s left, the other to the participant’s right, with a distance of ~40 cm between them. The participants placed their hands next to the near LEDs (the left hand next to the left LED and the right hand next to the right LED), with a maximum distance of 1 cm between the LED and the metacarpophalangeal joint of the index finger. The fixation LED was placed equidistantly from near and far LEDs and equidistantly from the left and right LEDs, at a distance of 65 cm in front of the body midline.

A trial started with the illumination of the fixation LED. After 500 ms, the nociceptive stimulus was applied either unilaterally, on the left or the right hand, or bilaterally, i.e. on both hands simultaneously. After an interval of 200 ms, the nociceptive stimulus was then followed by a pair of visual stimuli of 5 ms duration each, either presented near or far from the trunk, depending on the block. This interval was chosen based on previous studies [46] in which we showed that a 200 ms interval was the most efficient, as compared to 400 and 600 ms, for the nociceptive cue to impact the processing of the visual targets in the TOJ task. Ten possible time intervals (SOAs, i.e. stimulus onset asynchronies) were used between the two visual stimuli: ± 200, ±90, ±55, ±30, ±10 ms (negative values indicate that the left LED was illuminated first). Participants were instructed to keep their gaze at the fixation point during the whole trial. In half of the blocks they reported verbally which of the two visual stimuli they perceived as appearing first, while they reported which visual stimulus they perceived as appearing second in the other half of the blocks (by answering ‘left’ or ‘right’). By using these two response modalities and by averaging the associated data before the statistical analysis, the influence of decisional biases that could be mistaken for genuine perceptual spatial biases can be minimized (see e.g. [25, 47, 48] for a discussion). The participants didn’t receive any specific instruction regarding response speed and no feedback regarding the accuracy of their performance was given. Illumination of the fixation point was switched off as soon as the response was encoded by the experimenter and the next trial started 2000 ms later.

The experiment was composed of four blocks resulting from the combination of the position of the visual stimuli (near vs. far) and the response modality (‘which is first’ vs. ‘which is second’). The order of the blocks was randomized. Each block consisted of three series of 20 trials, one for each cue condition: unilateral left vs. unilateral right vs. bilateral nociceptive stimuli. The trials of the three series were randomly and equiprobably intermixed. Within each of the series, the SOA that was actually presented out of the 10 possible SOAs at one trial was determined online trial by trial according to the adaptive PSI procedure [49], i.e. based on the participant’s performance on all previous trials within one cue condition (implemented trough the Palamedes Toolbox, [50]).

Levels of perceived intensity of the nociceptive stimuli were assessed (on a scale from 0 to 10, with 0 = no sensation and 10 = very intense sensation) after each block, to ensure they were still perceived and that their intensities were rated as equivalent for both hands. If these criteria were not met, the intensities were adapted, or the electrodes were displaced and the absolute threshold measurements restarted (see [43] for details). A rest period between the blocks was possible when requested. Duration of the whole experiment was approximately 45 min. As changes in perceived intensities and the subsequent adaptation of intensities between blocks were completely random and unrelated to one particular experimental condition, stimulus intensities were characterized for each participant and each hand by the highest intensity of current adjusted during the experiment for further analyses.

#### 2.1.4 Measures

We consider two measures to assess the performance of the participants in the TOJ task: the point of subjective simultaneity (PSS) and the slope. More in detail, we estimated these measures as the α and β parameters of a logistic function i.e. $f(x)=\frac{1}{1+\exp\left(-\beta (x-\alpha )\right)}$. The α defines the threshold of the function, which, in our study, corresponds to the SOA at which the two visual stimuli are perceived as occurring first equally often (i.e. the 0.5 criterion on the ordinate). Accordingly, this measure corresponds to the PSS which is defined as the amount of time one stimulus has to precede or follow the other in order for the two stimuli to be perceived as occurring simultaneously [51]. The β parameter defines the slope of the logistic function, which describes the noisiness of the results and can be related to the precision of participants’ responses during the experiment [52]. The slope of the psychometric function is often used to derive the just noticeable difference (JND) in typical TOJ experiments. In order to estimate the logistic function we used the adaptive PSI method [49], in which the psychometric curve and its parameters are estimated at each trial. This specific method adapts the experimental procedure and the presented SOAs according to the performance of the participant on all the previous trials. This method is based on an algorithm that adopts a Bayesian framework, with the ultimate goal to estimate the parameters of interest without probing extensively all the SOAs (see [46, 52] for a more detailed description and the advantages of using the PSI method in TOJ experiments).

The average of the PSS values for left sided cues and the PSS values (multiplied by -1) for right sided cues was calculated to derive a unilateral cue condition for each participant and each experimental condition. For the unilateral cue conditions, the proportion of trials in which the visual stimulus presented in the cued side of space was reported as appearing first was plotted as a function of SOA. For the bilateral cue conditions, it was the proportion of trials in which the left visual stimulus was reported as appearing first that was plotted as a function of SOA.

#### 2.1.5 Data analysis

The means across participants of the maximal intensity of the nociceptive stimuli between both hands were compared using a paired-samples t-test. Regarding TOJ data, before statistical analyses data from the two response modalities (‘which is first’ vs. ‘which is second?’) were merged to reduce potential response biases. First, simple t-tests were performed, comparing each PSS value to 0, with the aim to characterize potential shifts in TOJs to one side of space in the different experimental conditions. The differences between PSS and slope values across conditions were tested using an analysis of variance (ANOVA) for repeated measures with *cue condition* (unilateral vs. bilateral) and *visual stimuli position* (near vs. far) as within-participant factors. Greenhouse-Geisser corrections of degrees of freedom and contrast analyses were used when necessary. Significance level was set at p ≤ .05. Effect sizes were measured using Cohen’s d for t-tests or partial Eta squared for ANOVAs.

### 2.2 Experiment 2

#### 2.2.1 Participants

In total, 30 participants were recruited in August 2015 through advertisements on the internet. Four participants dropped out before the testing session and we finally tested 26 participants. The experiment took place between August and September 2015 at the Institute of Neuroscience of the Université catholique de Louvain, Brussels. Three participants were excluded before data analysis, one because of a technical problem and two because of difficulties in perceiving the nociceptive stimuli. The remaining 23 participants (17 women) had a mean age of 24.1 ± 4.46 years (range: 18–33 years). Exclusion criteria were the same as in Experiment 1. According to the Flanders questionnaire, 21 participants were right-handed and two left-handed. The experimental procedure was approved by the local ethic committee (Commission d’Ethique Biomédicale Hospitalo-Facultaire de l’Université catholique de Louvain) in agreement with the latest version of the Declaration of Helsinki and was carried out in accordance with the corresponding guidelines and regulations. All participants signed a consent form prior to the experimental session. Participants received financial compensation for their participation.

#### 2.2.2 Stimuli and apparatus

Nociceptive stimuli were applied in the same manner as in Experiment 1. Stimulation intensities were also determined as in Experiment 1. All the participants described the sensation as pricking.

Visual stimuli were presented by means of the four same white LEDs as in Experiment 1 and a yellow LED served as fixation point.

#### 2.2.3 Procedure

**Fig 2:**
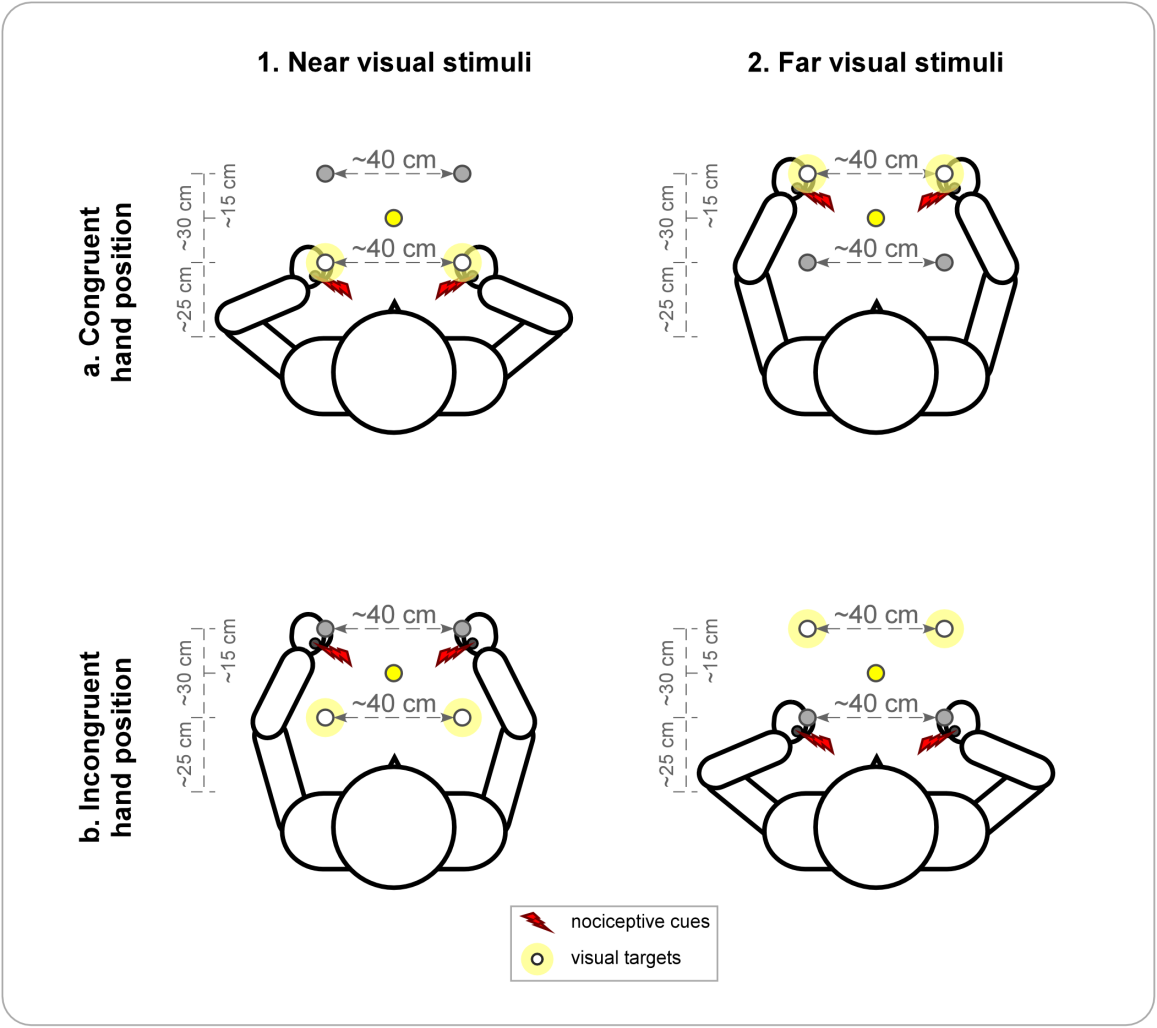
Design of Experiment 2. Visual stimuli are presented by means of two pairs of LEDs, one placed near and the other placed far from the trunk. The participants’ hands are positioned either close to the near LEDs or close to the far LEDs. In each block, participants perform the task only with one pair of LEDs, either *near* (1) or *far* (2) from the trunk, and the position of the hands is either *congruent* (1a & 2a) or *incongruent* (1b & 2b) relative to the position of the visual stimuli. The task-relevant pair is considered as the visual targets and illustrated by the white circles with a slight yellow halo. Nociceptive stimuli are illustrated by the red flashes and, depending on the trial, they were applied unilaterally either on the left or the right hand. A centrally-placed yellow LED, represented by the yellow circles, serves as fixation point.

The experimental set-up and procedure were similar to Experiment 1. Participants were sitting in front of a table, their heads stabilized with a chin-rest and their hands placed on the table, palms down. The LEDs were fixated on the table. Two of the white LEDs were placed at a distance of ~25 cm from the trunk (near visual stimuli), and two LEDs at a distance of ~55 cm from the trunk (i.e. ~30 cm from the near LEDs) (far visual stimuli). The distance between the left and right LED of each pair was ~40 cm. The fixation LED was placed equidistantly from the near and far LEDs and equidistantly from the left and right LEDs, at a distance of ~40 cm in front of the body midline. The participants placed their hands either next to the near LEDs or next to the far LEDs, depending on the block (Fig 2). The left hand was always placed next to the left LED and the right hand next to the right LED.

A trial started with the illumination of the fixation LED. After 500 ms, a nociceptive stimulus was applied either unilaterally on the left or unilaterally on the right hand. As compared to Experiment 1, we did not use bilaterally applied nociceptive stimuli anymore, since we showed in previous studies [46] and in Experiment 1 (see 3.1.2) that bilateral cues do not induce significant biases to any of the two sides of space. This allowed us to reduce the number of trials and therefore to double the number of conditions without extending the duration of the experiment excessively. After 200 ms, the nociceptive stimulus was followed by a pair of visual stimuli of 5 ms duration each, either presented near or far from the trunk, depending on the block. Accordingly, the hands were placed in a position that was either congruent or incongruent with the position of the pair of visual stimuli on which the TOJ task was performed (congruent: TOJ on near visual stimuli and hands next to near LEDs, TOJ on far visual stimuli and hands next to far LEDs; incongruent: TOJ on near visual stimuli and hands next to far LEDs, TOJ on far visual stimuli and hands next to near LEDs). Twenty possible SOAs were used between the two visual stimuli: ±200, ± 145, ±90, ± 75, ± 60, ±45, ±30, ±15, ± 10, ± 5 ms (negative values indicate that the left LED was illuminated first). Since we used an adaptive method where only SOAs for which one might expect to gain the most information about the parameters of interest are presented [52], we decided to include more possible SOAs than in Experiment 1, to reduce the risk of errors due to inappropriately chosen SOA levels. Participants were instructed to keep their gaze at the fixation point during the whole trial. Depending on the block, they either reported which of the two visual stimuli they perceived as appearing first, or which of the stimuli they perceived as appearing second. No specific instruction regarding response speed and no feedback regarding the accuracy of the performance were given. Illumination of the fixation point was switched off as soon as the response was encoded by the experimenter and the next trial started 2000 ms later.

The experiment was composed of eight blocks resulting from the combination of the position of the visual stimuli (near vs. far), the congruency of the position of the hands relative to the visual stimuli (congruent vs. incongruent) and the response modalities (‘which is first’ vs. ‘which is second’). The order of the blocks was randomized. Each block consisted of two series of 20 trials, one for each cueing condition: unilateral left vs. unilateral right. The trials of the two series were randomly and equiprobably intermixed. Within each of the series, the presented SOAs were chosen according to the adaptive PSI procedure [49], as explained for Experiment 1.

As in Experiment 1, levels of perceived intensity of the nociceptive stimuli were assessed after each block and the intensities adapted or the electrodes displaced and the threshold measurements restarted if necessary. A rest period between the blocks was possible when requested. Duration of the whole experiment was approximately 60 min.

#### 2.2.4 Measures

The measures were the same as for Experiment 1. PSS values for left sided cues and PSS values (multiplied by -1) for right sided cues were again averaged. The proportion of trials in which the visual stimulus presented in the cued side of space was reported as appearing first was plotted as a function of SOA.

#### 2.2.5 Data analysis

Analyses were similar to Experiment 1. The maximal intensity of the nociceptive stimuli between both hands was compared using a paired-samples t-test. Before statistical analyses of the TOJ, data from the two responses (‘which is first’ and ‘which is second?’) were merged. Each PSS value was compared to 0 using simple t-tests. An ANOVA for repeated measures was performed with *visual stimuli position* (near vs. far) and *hand congruency* (congruent vs. incongruent) as within-participant factors, for both PSS and slope.

## 3. Results

### 3.1 Experiment 1

#### 3.1.1 Intensity of nociceptive stimuli

The mean of the maximal intensities was 0.35±0.08 mA for nociceptive stimuli applied to the left hand and 0.35±0.09 for nociceptive stimuli applied to the right hand (no significant difference: *t*(16) = -0.39, *p* = 0.97). These intensities are in the range of values that have been shown to selectively activate skin nociceptors in previous studies [42, 44, 45].

#### 3.1.2 PSS

**Fig 3:**
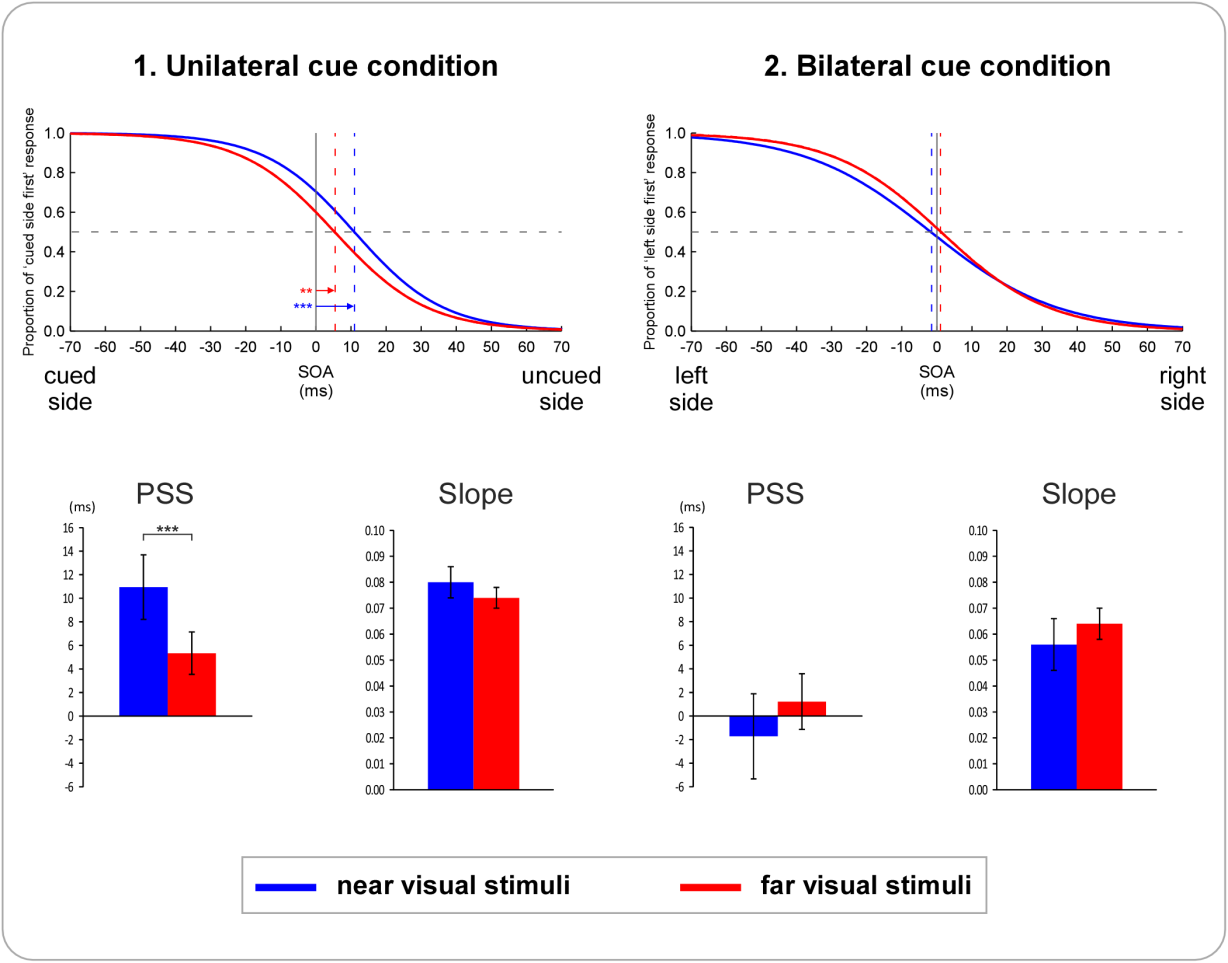
Results of Experiment 1. The figure illustrates the averaged results of the 17 participants. Data from left-sided nociceptive cue conditions and right-sided nociceptive cue conditions were averaged into a unilateral cue condition. The upper part depicts the fitted logistic functions for the (1) *unilateral* and the (2) *bilateral* cue condition. For the unilateral cue condition (1), the x-axis represents different hypothetical stimulus onset asynchronies (SOAs) between the two visual stimuli: negative values indicate that the visual stimulus occurring in the cued side of space was presented first, while positive values indicate that the visual stimulus occurring in the uncued side of space was presented first. The y-axis represents the proportion of trials in which the participants perceived the visual stimulus presented in the cued side of space as occurring first. For the bilateral cue condition (2), negative SOA values on the x-axis indicate that the visual stimulus occurring in the left side of space was presented first, while positive values indicate that the visual stimulus occurring in the right side of space was presented first. The y-axis represents the proportion of trials in which the participants perceived the stimulus presented in the left side of space as occurring first. In both (1) and (2), blue curves represent the conditions in which visual stimuli were presented near the trunk, with the corresponding PSS values indicated by the blue vertical dashed lines. Red curves represent the conditions in which visual stimuli were presented far from the trunk, with the corresponding PSS values indicated by the red vertical dashed lines. The arrows in (1) indicate the PSS values significantly different from zero. The curves in the unilateral cue condition are significantly shifted to the uncued side of space, indicating that visual stimuli presented in the uncued side of space had to be presented several ms before the stimuli presented in the cued side of space to have the chance to be perceived as occurring first equally often. There are no significant shifts to either side of space in the bilateral condition. The lower part illustrates the mean PSS and slope values, for both the unilateral (1) and the bilateral (2) cue condition. Significant differences are indicated with asterisks (* p ≤ .05, ** p ≤ .01, *** p ≤ .001). PSS values were significantly different between visual TOJs performed near vs. far from the trunk, but only in the unilateral cue condition (asterisks in the bar graphs illustrate the p-value of the contrast resulting from the significant interaction between *cue condition* and *visual stimuli position* factors). Error bars represent the 95% confidence intervals adapted according to the method of Cousineau [53].

**Fig 4:**
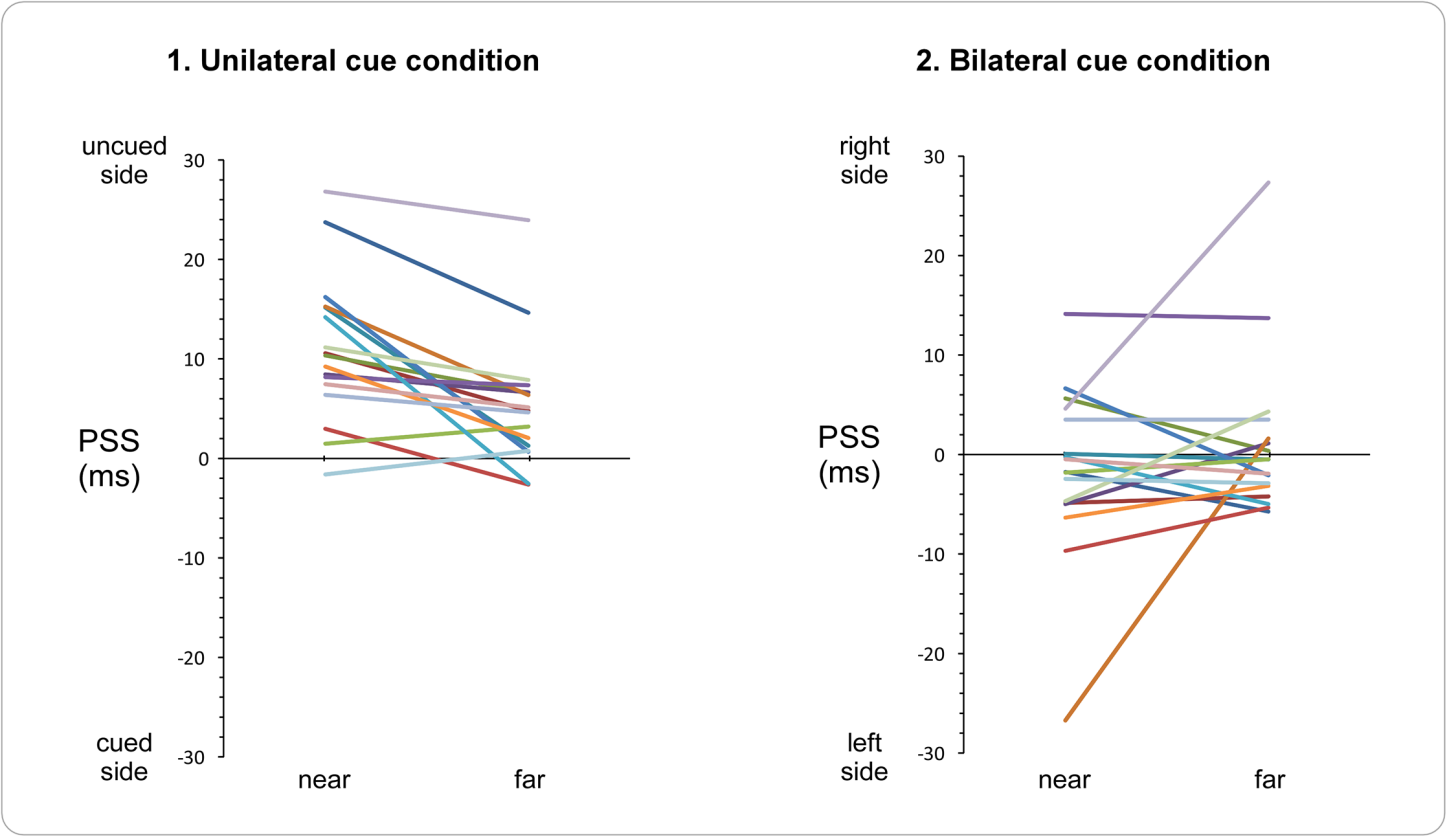
Individual PSS values for Experiment 1. The left graphic illustrates the PSS values for the *unilateral cue* conditions (1), and the right graphic the PSS values for the *bilateral cue* conditions (2). Within each graphic, the left side illustrate the PSS values of the tasks performed with the near visual stimuli, the right side those with the far visual stimuli. Each color line represents one of the 17 participants who participated in the task. For the unilateral cue conditions (1) there is almost systematically a shift of the PSS to the uncued side, and these shifts are also almost systematically larger for near than for far visual stimuli. For the bilateral (2) cue conditions, shifts in PSS seem rather random, either to the left or to the right side, and there is also no systematic difference between near and far visual stimuli.

Results of Experiment 1 are illustrated in Figs 3 and 4. Simple t-tests showed that PSS values were significantly different from zero for the unilateral cue conditions, for both the near (*t*(16) = 6.19, *p≤* 0.001, d = 1.50) and the far (*t*(16) = 3.46, *p* = 0.003, d = 0.84) visual stimuli. The visual stimuli appearing in the uncued side of space had thus to be presented significantly earlier than stimuli appearing in the cued side of space to have the chance to be perceived as occurring simultaneously. On the contrary, PSS values for the bilateral cue conditions were never significantly different from zero (all *t*(16)≤ 0.61, *p*≥ 0.42). These results suggest that unilateral cues can induce spatial biases.

The ANOVA showed a significant main effect of *cue condition* (*F*(1,16) = 24.37, p*≤* 0.001, *Ƞ^2^p* = 0.60) and a significant interaction between *cue condition* and *visual stimuli position* (*F*(1,16) = 11.93, *p* = 0.003, *Ƞ^2^p* = 0.43). There was no significant main effect of the factor *visual stimuli position* (*F*(1,16) = 0.84, *p* = 0.37, *Ƞ^2^p* = 0.05). Contrast analyses revealed that there was a significant difference between the near and the far visual stimuli position for the unilateral cue condition (*F*(1,16) = 16.66, *p* = 0.001, *Ƞ^2^p* = 0.51), whereas such a difference was not significant for the bilateral cue condition (*F*(1,16) = 1.61, *p* = 0.22, *Ƞ^2^p* = 0.09). This suggests that the spatial biases induced by unilateral nociceptive cues are significantly bigger when visual stimuli are presented near (M = 10.95, SD = 7.29) than when they are presented far (M = 5.34, SD = 6.36) from the trunk. This interaction is illustrated in Fig 4, which shows at the individual level a nearly systematic difference between TOJ performed on near vs. far visual stimuli only in the unilateral cue condition.

#### 3.1.3 Slope

The ANOVA revealed a significant main effect of *cue condition* (*F*(1,16) = 25.66, *p≤* 0.001, *Ƞ^2^p* = 0.62). There was neither a significant main effect of *visual stimuli position* (*F*(1,16) = 0.04, *p* = 0.84, *Ƞ^2^p* = 0.04) nor a significant *cue condition* x *visual stimuli position* interaction (*F*(1,16) = 2.79, *p* = 0.11, *Ƞ^2^p* = 0.15). Slopes were in general steeper in the unilateral cue conditions than in the bilateral cue conditions, indicating that judgments were less noisy, i.e. less variable, when unilateral nociceptive cues were presented. This corroborates the PSS results reported in 3.1.2, since steeper slopes suggest that judgments were more systematically biased by unilateral cues and thus more precise than in the bilateral cue conditions.

### 3.2 Experiment 2

#### 3.2.1 Intensity of nociceptive stimuli

The mean of the maximal intensities was 0.31±0.08 for the nociceptive stimuli applied on the left hand and 0.30±0.1 for nociceptive stimuli applied on the right hand (difference not significant: *t*(22) = 0.63, *p* = 0.53).

#### 3.2.2 PSS

**Fig 5:**
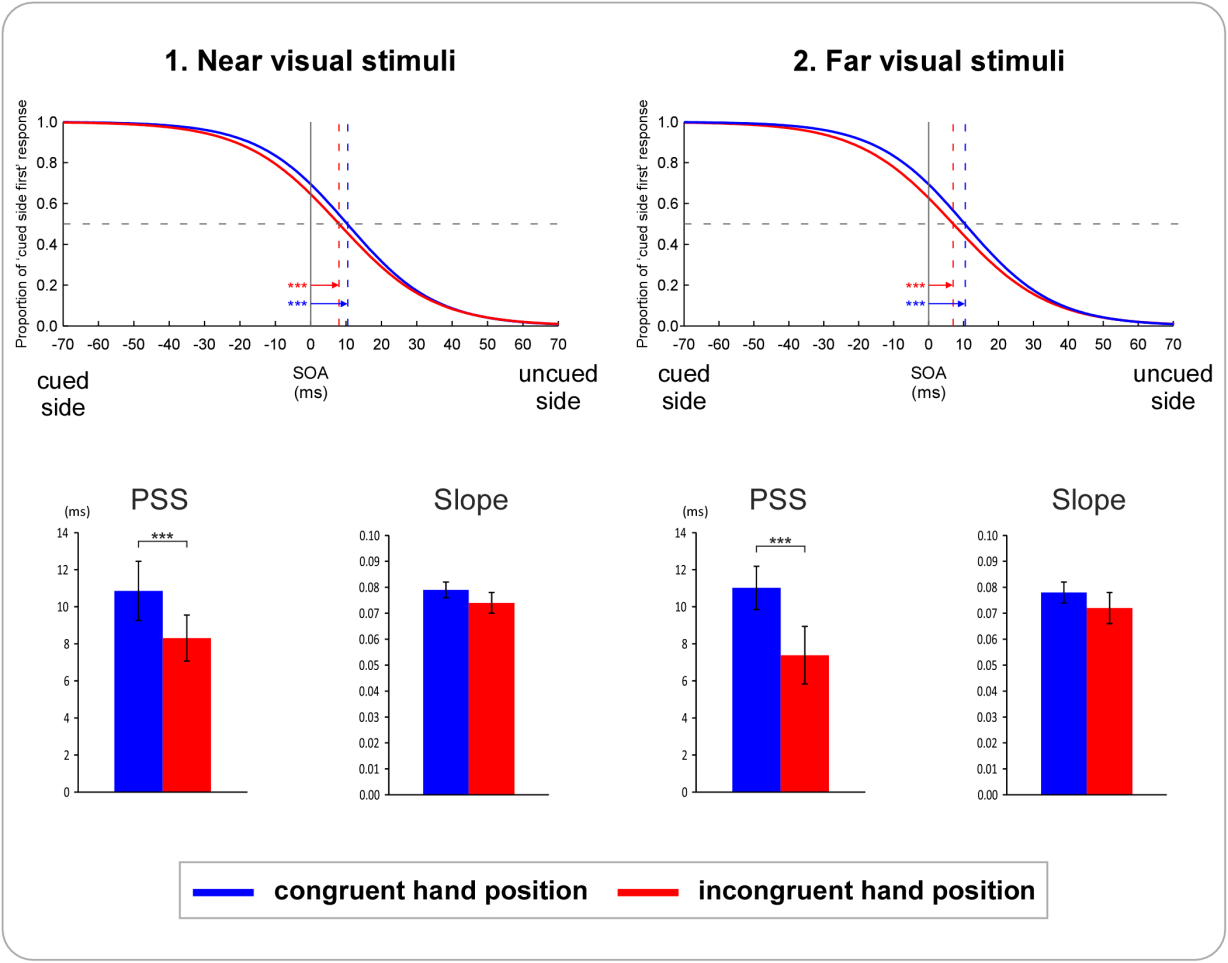
Results of Experiment 2. The figure illustrates the averaged results of the 23 participants. Data from left-sided cue conditions and right-sided cue conditions were averaged into a unilateral cue condition. The upper part depicts the fitted logistic functions for the (1) near *visual stimuli* and the (2) far *visual stimuli* condition. The x-axis represents different hypothetical stimulus onset asynchronies (SOAs) between the two visual stimuli: negative values indicate that the visual stimulus occurring in the cued side of space was presented first, while positive values indicate that the visual stimulus occurring in the uncued side of space was presented first. The y-axis represents the proportion of trials in which the participants perceived the visual stimulus presented in the cued side of space as occurring first. Blue curves represent the conditions in which the participants’ hands were placed in a congruent position relative to the visual stimuli, with the corresponding PSS values indicated by the blue vertical dashed lines. Red curves represent the conditions in which the participants’ hands were placed in an incongruent position relative to the visual stimuli, with the corresponding PSS values indicated by the red vertical dashed lines. The arrows indicate the PSS values significantly different from zero. In both (1) and (2) the curves are significantly shifted to the uncued side of space, indicating that visual stimuli presented in the uncued side of space had to be presented several ms before the stimuli presented in the cued side of space to have the chance to be perceived as occurring first equally often. The lower part illustrates the mean PSS and slope values, for both the near visual stimuli (1) and the far visual stimuli (2) condition. Significant differences are indicated with asterisks (* p ≤ .05, ** p ≤ .01, *** p ≤ .001). PSS values were significantly different between visual TOJs performed in the congruent vs. incongruent conditions, for TOJs performed on both near and far visual stimuli (asterisks in the bar graphs illustrate the p-value of the significant main effect of the factor *hand congruency*). Error bars represent the 95% confidence intervals adapted according to the method of Cousineau [53].

Results of Experiment 2 are illustrated in Fig 5. The PSS values of the four condition were all significantly different from zero (near visual stimuli, hands in congruent position: *t*(22) = 5.96, *p≤* 0.001, d = 1.24; near visual stimuli, hands in incongruent position: *t*(22) = 5.08, *p≤* 0.001, d = 1.06; far visual stimuli, hands in congruent position: *t*(22) = 6.02, *p≤* 0.001, d = 1.26; far visual stimuli, hands in incongruent position: *t*(22) = 5.52, *p≤* 0.001, d = 1.15). Judgments were biased to the advantage of visual stimuli presented in the same side of space as the nociceptive stimuli.

The ANOVA showed a significant main effect of *hand congruency* (*F*(1,22) = 16.80, *p≤* 0.001, *Ƞ^2^p* = 0.43). Neither the main effect of *visual stimuli position* nor the interaction between *hand congruency* and *visual stimuli position* was significant (all *F*≤ 0.36, *p*≥ 0.55). These results suggest that the spatial biases induced by nociceptive lateralized cues were significantly more important when the hands were placed in a congruent position (M = 10.94, SD = 8.33) than when the hands were placed in an incongruent position (M = 7.85, SD = 6.64) with regard to the visual stimuli.

#### 3.2.3 Slope

The ANOVA revealed no significant main effects and no significant interaction (all *F*≤ 3.08, *p*≥ 0.09).

## 4. Discussion

The aim of the present studies was to demonstrate that the perception of visual space can be affected by somatosensory sensations felt on a limb occupying one particular location in space. Such crossmodal interactions are especially important when these somatosensory sensations indicate potential body damage and when the source of this potential damage has to be located in the external world. Concretely, by using TOJ tasks, we aimed at inducing biases in the perception of the visual stimuli elicited by nociceptive stimuli applied on one of the hands. Interpretation of performance during TOJ is based on the theory of prior entry [54] according to which stimuli on which we focus our attention are perceived earlier than unattended ones. As a consequence, to get the chance to be perceived as appearing at the same time as the attended stimulus, the unattended stimulus has to be presented before the attended stimulus. In TOJ tasks, the time interval at which both stimuli are perceived as occurring first equally often (i.e. the PSS) can thus be used to index shifts in attention to one of the two stimuli. In our first TOJ experiment, participants judged the temporal order of pairs of lateralized visual stimuli, either presented near or far from the trunk, which were preceded by nociceptive stimuli applied either on one single hand, i.e. unilaterally or on both hands simultaneously, i.e. bilaterally. The participants’ hands were always placed next to the visual stimuli presented near the trunk. Results showed that the presence of unilateral nociceptive stimuli biased temporal order perception of the visual stimuli, with TOJs prioritizing the visual stimulus that was presented in the same side of space as the nociceptive stimuli. More precisely, visual stimuli presented in the uncued side of space had to be presented several ms before the visual stimuli presented in the cued side of space to have the chance to be perceived as occurring first equally often. When both hands were stimulated simultaneously with the nociceptive stimuli, no significant judgment biases to any of the two sides of space were observed, as attention was probably not shifted in one particular direction. Although biases were significant in the unilateral cue condition for both near and far visual stimuli, the magnitude of these biases was significantly larger for visual stimuli presented near the trunk than for visual stimuli presented farther away. In a second experiment, we tested whether this effect of the proximity between the nociceptive and visual stimuli was determined by the close distance of the visual stimuli to the hand on which the nociceptive stimuli were applied or rather by the general proximity to the body as a whole (and probably based on the trunk as reference). To this aim we added conditions in which the participants’ hands were also placed next to the visual stimuli presented farther away from the trunk. Manipulating both the position of the visual stimuli and the position of the participants’ hands allowed us to compare conditions in which the position of the hands was either congruent or incongruent with regard to the visual stimuli. The results revealed significant biases to the advantage of the visual stimuli presented in the same side of space as the stimulated hand in all conditions, but with a significantly larger effect when the hands were placed in a congruent position with regard to the visual stimuli. This suggests that the observed crossmodal effects between nociceptive and visual stimuli are rather related to the proximity of the visual stimuli to the hand on which the nociceptive stimuli are applied.

These findings give further support to the hypothesis that nociceptive and visual inputs are integrated to build a coherent multisensory representation of the body and the space immediately surrounding it [14, 55]. Such multisensory representation would act as coordinate system to code the spatial position of nociceptive stimuli on the body surface and the position of non-somatic, i.e. visual, stimuli immediately surrounding the body part on which the nociceptive stimuli are applied, that is, the body area potentially being damaged. Indeed, while interacting with proximal visual stimuli is of primary importance to optimize the manipulation of innocuous objects, it is even more important for optimizing defensive behaviors against potentially noxious objects [14, 29]. Detecting physical threat is first of all a priority for survival, as illustrated, for instance, by individuals suffering from congenital analgesia, a condition characterized by an inability to feel pain, mainly due to a genetic mutation affecting voltage-gated sodium channels that are expressed in nociceptive neurons (see [56, 57]). Due to the absence of pain perception, these individuals are exposed to important body damages since they do not develop any sense of danger. It is furthermore also important to monitor the space around the body to avoid stimuli that can induce pain, or, when a limb is already in pain, to avoid increasing the pain through the contact with external objects. This can be illustrated, for instance, by patients suffering from complex regional pain syndrome (CRPS), a chronic pain disorder predominantly characterized by sensory, trophic, motor and vegetative symptoms. Interestingly, deficits in perceiving and representing their own body, but also the visual external space have been reported in these patients (for a review see [14, 15]). Moseley et al. [58] for instance showed that the temporal order of pairs of tactile stimuli applied on the hands is judged to the disadvantage of tactile stimuli applied on the painful hand. When the hands were however crossed over the sagittal body-midline, patients’ perceptual judgments were made to the disadvantage of the non-painful hand, now placed in the space previously occupied by the painful hand. TOJ performances of CRPS patients were thus not determined by the painful limb, but rather by the side of space in which that limb normally resides. These results suggest that pain can change other somatic sensations according to a personal reference frame that interacts also with proprioception, i.e. the relative position of the limbs in external space. Furthermore, Bultitude et al. [59] recently showed that biases in TOJ can also affect the perception of external, i.e. visual, stimuli, to the detriment of visual stimuli presented in the same side of space as the painful limb. These latter results show that the perception of the space external to the body can also be impaired in CRPS. Such deficits in spatial perception observed in CRPS have been suggested to result from a maladaptive neuroplasticity consecutive to (implicit) behavioral strategies developed by the patients, aimed at avoiding an increase of pain of the pathological limb [60]. Finally, there are preliminary results suggesting that acting on visuospatial perception, for instance, by visual displacement with prismatic goggles combined with sensori-motor coordination, can reduce pain and other CRPS-related symptoms (for a review see [61]). Clarifying how nociceptive and visual inputs interact is therefore also of primary importance for the development of new rehabilitation techniques to reduce chronic pain.

Our results complement those observed by De Paepe and colleagues [24, 27], who demonstrated in series of TOJ experiments that nociception, like touch, is also likely to be integrated in a common, peripersonal, reference frame, by showing that visual inputs presented in the vicinity of the body can affect how we perceive nociceptive stimuli. Here we extend these results, by demonstrating that nociception, for its part, can also influence visual perception, suggesting that the way we perceive and represent our near visual surrounding is built on strict links with somatosensory, i.e. nociceptive perception.

Furthermore, we also showed that these crossmodal spatial interactions between nociception and vision seem to partially rely on a spatial representation of a specific body part, that is, more specifically in this study, the hand, that extends slightly in its external surrounding. Independently of the distance of the visual stimuli and the hands from the trunk, crossmodal influence was largest when visual stimuli were presented near the hands. This finding is in line with studies in the tactile modality suggesting that peripersonal reference frames for spatial perception operate in limb-centered coordinates. Indeed, animal studies revealed the existence of bimodal neurons, for instance in the PMv, associating a tactile receptive field on a specific body part and a visual receptive field in the same spatial area, anchored to the tactile receptive field (e.g. [7, 8]). Importantly, it has been shown that the visual receptive field moves with the corresponding tactile one, independently of the gaze direction and eye movement, (e.g. [30, 31, 32, 33]). In humans, evidence comes from studies suggesting that crossmodal visuo-tactile extinction arises in body part-centered rather than trunk or retinal-centered coordinates. In other words, a tactile stimulus applied on a left limb can be extinguished by a visual stimulus presented in the contralateral space, but on condition that this visual stimulus occurs close to the homologous contralateral limb (e.g. [62, 63]). In addition, recent neuroimaging studies in healthy participants showed that the cortical activity in premotor and parietal areas is differently affected by visual stimuli presented near the hand as compared to visual stimuli presented in other spatial locations [64, 65]. Contrary to visuo-tactile interactions [13, 66] however, the neural bases of nociceptive-visual interaction are muss less systematically explored. For instance, to our knowledge, there is only one study that investigated the existence of bimodal neurons in monkeys and which described neurons in the inferior parietal lobe responding to both thermo-nociceptive stimuli and visual stimuli that approach the stimulated body part [38]. In humans, neuroimaging and neurophysiological studies have shown that nociceptive stimulations activate a broad network of cortical areas, but recent investigations have also demonstrated that almost none of these cortical areas are specifically involved in nociceptive processing, as they are able to respond to other somatic and, even, non-somatic stimuli [67]. Therefore, it has been proposed that the activity of these cortical areas responding to nociceptive stimuli represent the activity of a multimodal system which prioritizes the processing of stimuli that can impact body homeostasis, such as nociceptive and proximal visual stimuli [67]. Interestingly, this cortical network responding to nociceptive stimuli comprises regions, such as parietal areas, that have been described to be involved in multisensory interaction between visual and tactile stimuli in both non-human (e.g. [34, 35]) and human (e.g. [68, 69–71]) primates. A recent neuroimaging study for example showed that changing the hand posture during the application of mechanical nociceptive stimuli modulated the activity of the parietal cortex [72]. Furthermore, CRPS patients, who are characterized by cognitive difficulties affecting their ability to represent their body and space [15, 59], also show significant functional reorganization in sensori-motor and parietal areas [73]. We might therefore speculate that, as already described for touch, the parietal cortex, among other areas, plays an important role in the mechanisms underlying the interaction between nociceptive and proximal visual stimuli [74].

Since we did not control for gaze shifts throughout the experiments, it could be argued that our results could be explained by shifts in overt attention to the hand on which the nociceptive stimulus was applied which in turn would increase the foveal acuity of the spatially congruent visual stimuli, rather than by an interaction between nociception and vision within a peripersonal representation of space. This seems however unlikely, since, due to the slow conduction velocity of the Aδ-fibers that convey the nociceptive inputs induced by IES [75], the nociceptive input takes at least ~150 ms to reach the cortical level (see also [46]). Accordingly, since the time interval between the onset of the nociceptive stimulus and the first visual stimulus was 200 ms, the time interval between the respective arrivals of the nociceptive and visual inputs at the cortical level would be inferior to the duration of a saccade [75]. Furthermore, to minimize the possibility of gaze shifts during a trial, the fixation LED was switched off after the response of the participant was encoded and switched on again before the next trial, which allowed recapturing the participant’s attention towards the fixation.

It is important to note that the nociceptive stimuli used in the present studies were not necessarily perceived as painful by the participants, as the primary objective was to use a stimulus that specifically and selectively activates nociceptive pathways. Nevertheless, in future studies, it could be interesting to consider the use of nociceptive stimuli that induce sensations that are clearly perceived as painful, in order to investigate the relationship between the painfulness of a nociceptive stimulus and the efficiency or the extent of crossmodal attention shifts. However, one would also have to consider that possible changes in bias with more painful stimuli could be due to other confounding factors than simply a change from a non-painful to a painful percept, such as the emotional valence of the stimulus and its intensity. The same comment could be addressed regarding the emotional value of the visual stimuli. The extra-somatic stimuli used in the present experiments consisted indeed in neutral light flashes, and not in visual images representing physical threats (e.g. a needle). It should however be noted that, because nociceptive stimuli were actually not painful and the visual stimuli not threatening, the nociceptive-visual interaction observed in the present experiments seems to rely on attentional mechanisms acting independently of voluntary control and of the meaning and relevance of the stimuli.

In conclusion, the present experiments further demonstrated that a nociceptive input can be integrated in a spatial representation of the limb on which the nociceptive stimulus is applied that associates the limb itself and the space immediately surrounding it, in order to impact the perception of extra-somatic stimuli occurring in the immediate vicinity of that limb.
